# Social Perception of Illusory Faces: Effects of Width-to-Height Ratio, Chin Shape, and Eye–Mouth Distance

**DOI:** 10.3390/bs15070958

**Published:** 2025-07-15

**Authors:** Yaqi He, Wenhui Tan, Yuhan Dai, Yuxin Duan, Xin Liu, Guomei Zhou

**Affiliations:** Department of Psychology, Sun Yat-sen University, Guangzhou 510006, China

**Keywords:** illusory face, pareidolia, width-to-height ratio, chin shape, eye–mouth distance, social evaluation

## Abstract

While real face features are known to influence social evaluations, the social perception of illusory faces remains largely unexplored despite neural similarities to real faces. This study aimed to fill this gap by manipulating the width-to-height ratio, chin shape, and eye–mouth distance of illusory faces and assessing their effects on perceived gender, cuteness, trustworthiness, dominance, attractiveness, and emotion. Key findings include the following: (1) high width-to-height ratios significantly boosted attractiveness for female participants but not for male participants; (2) round chins consistently enhanced perceptions of masculinity, cuteness, attractiveness, and trustworthiness; (3) eye–mouth distance was found to affect emotional perception. This research offers crucial experimental insights into the determinants of social evaluations for illusory faces.

## 1. Introduction

The human tendency to perceive faces in everyday objects, such as a cut tomato, is known as face pareidolia or illusory face (Palmer & Clifford, 2020; Taubert et al., 2017; Wardle et al., 2020). This phenomenon is often considered a natural “error” in our face detection system. Importantly, these illusory faces can convey social characteristics, much like real faces. For instance, Wardle et al. (2022) found that participants tended to perceive illusory faces as male, happy, and younger. While extensive research exists on the social evaluation of real faces, focusing on dimensions like dominance, attractiveness, and credibility (Oosterhof & Todorov, 2008; Todorov et al., 2015), fewer studies have explored the social evaluation of distorted faces. This study aims to bridge that gap by investigating which physical features contribute to these social evaluations in illusory faces, including the dimensions of dominance, attractiveness, and trustworthiness, as well as gender, emotion, and cuteness.

Research on real faces has identified the facial width-to-height ratio (the distance between the left and right zygion (bizygomatic width) divided by the distance between the upper lip and mid-brow) as a significant feature influencing social perceptions. For instance, Weston et al. (2007) found greater width-to-height ratios in men than in women. Carré et al. (2009) demonstrated a positive correlation between facial width-to-height ratio and estimated aggression and dominance. The width-to-height ratio also relates to trustworthiness and attractiveness, with higher width-to-height ratios in male faces often perceived as less trustworthy (Costa et al., 2017; Stirrat & Perrett, 2010) and less attractiveness (Durkee & Ayers, 2021; Stirrat & Perrett, 2010).

Beyond the width-to-height ratio, the chin is another crucial facial feature that influences gender and personality impressions. The chin, a unique human characteristic (Schwartz & Tattersall, 2000), contributes to judgments of trustworthiness (Todorov et al., 2008). Specifically, a wide chin is generally considered one of the features that increase facial trustworthiness, while a thin chin may be associated with lower trustworthiness. Manipulating chin shape has shown that a round chin can make a face appear happier, while an angular chin can convey anger (Becker et al., 2007). Glocker et al. (2009) also demonstrated that a “baby schema,” including a round face, enhances perceived cuteness. Additionally, Almanza-Sepúlveda et al. (2018) found that babies with rounder heads are perceived as cuter. Anthropological studies have long noted gender differences in chin shape, with men typically having wider chins and women having more pointed chins (Bass & Riggio, 2006; Byers, 2016). These findings underscore the importance of including chin shape as a facial factor in our study.

The distance between the eyes and mouth is another key element of facial composition, often associated with the “golden ratio.” Studies show that this distance affects social evaluations. For example, a smaller eye–mouth distance can intensify perceived anger, while a larger distance can heighten perceived sadness (Neth & Martinez, 2009). Glocker et al. (2009) found that a smaller eye–mouth distance in infant faces increased perceived cuteness and attractiveness. In virtual character design, eye and mouth proportions consistent with baby schema significantly enhance character credibility (Hu, 2024). Research on adult female faces suggests a “golden ratio”, where an eye–mouth distance accounting for 36% of the face’s vertical length is most attractive (Pallett et al., 2010). For Chinese individuals, this optimal ratio is 33% (Lin & Zhou, 2021). These studies provide strong justification for incorporating eye–mouth distance as a facial factor in our research.

The human brain possesses dedicated neural circuits for face processing, particularly for social attributes, including the fusiform face area, occipital face area, and inferior frontal gyrus (Kanwisher & Yovel, 2006). Research indicates that both real and misperceived faces activate these regions (Liu et al., 2014). Given this neural commonality, we speculate that the social evaluation of distorted faces may also be influenced by factors like width-to-height ratio, chin shape, and eye–mouth distance.

Furthermore, gender differences exist in the neural mechanisms that underly the processing of illusory faces. Proverbio and Galli (2016) found that women’s brains are more prone to anthropomorphizing objects, with face–object perception in women activating areas related to emotional and social information processing (e.g., right superior temporal lobe, posterior cingulate cortex, orbitofrontal cortex; Xiu et al., 2015). Men, conversely, show more activation in areas related to shape processing. Proverbio and Galli (2016) also reported that women rated face-like everyday objects significantly higher in “faceness” than men. After recognizing these clear gender differences, we have included gender as a factor in our study.

Building on this background, our study investigates the perception of gender, attractiveness, dominance, trustworthiness, cuteness, and emotion in illusory faces. We specifically examine how their width-to-height ratio (Experiment 1), chin shape (Experiment 2), and eye–mouth distance (Experiment 3) influence these social judgments. Consistent with previous findings on the width-to-height-ratio of real faces (Carré et al., 2009; Costa et al., 2017; Durkee & Ayers, 2021; Stirrat & Perrett, 2010), we hypothesize that a higher width-to-height ratio will be associated with greater dominance but lower perceived trustworthiness and attractiveness. In line with previous research on the chin shape of real faces (Almanza-Sepúlveda et al., 2018; Bass & Riggio, 2006; Becker et al., 2007; Byers, 2016; Glocker et al., 2009; Todorov et al., 2008), we expect that a round chin (vs. a pointed chin) will be associated with greater femaleness, cuteness, trustworthiness, and happiness, while being linked to lower perceived anger. Furthermore, building on previous work regarding the eye–mouth distance of real faces (Glocker et al., 2009; Hu, 2024; Lin & Zhou, 2021; Neth & Martinez, 2009; Pallett et al., 2010), we anticipate that a smaller eye–mouth distance will intensify perceived anger and increase perceived cuteness and trustworthiness. In contrast, a larger eye–mouth distance will heighten perceived sadness, while an optimal eye–mouth distance will enhance perceived attractiveness.

## 2. Experiment 1: Changing the Facial Width-to-Height Ratio

### 2.1. Methods

#### 2.1.1. Subjects

A G*Power 3.1.9.7 (Faul et al., 2009) analysis for a paired t-test, with an effect size (d) of 0.45, a significance level (α) of 0.05, and a desired statistical power (1 − β) of 0.80, indicated a required sample size of 41 participants. To ensure a balanced experimental design and maximize recruitment, we enrolled 54 participants (30 males, 24 females, aged 18–25) via advertisements. All participants reported normal or corrected-to-normal vision, had no face recognition difficulties, and were not psychology students. They voluntarily participated and received compensation upon completion.

#### 2.1.2. Experimental Materials

We selected 50 illusory face images from the 256 open-source images provided by Wardle et al. (2022), most of which were previously confirmed to evoke face pareidolia. These images were initially cropped to squares (400 × 400 pixels) and converted to grayscale.

**Figure 1:**
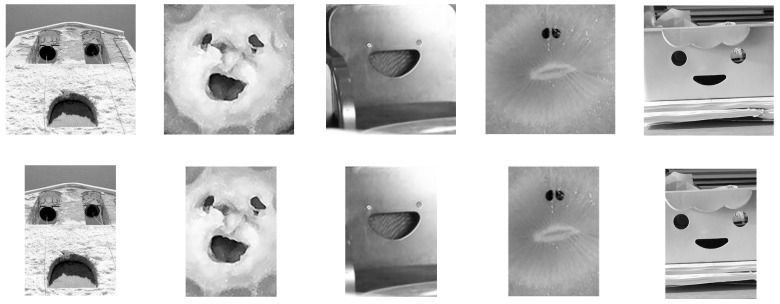
Examples of stimuli in Experiment 1. The images in the top row depict illusory faces with a high width-to-height ratio, adapted from Wardle et al. (2022), while the images in the bottom row show illusory faces with a low width-to-height ratio, which was cropped for this study.

To investigate the effect of the width-to-height ratio, we further manipulated these images using Photoshop. Due to variations in the original images (e.g., various face shapes and sizes, and most of them lacking clear zygions), it was difficult to measure the width (distance between the left and right zygion) and maintain absolute high or low width-to-height ratio values across all the images. Therefore, we used the original 400 × 400 pixels images as the high width-to-height ratio (*M* = 3.65, *SD* = 2.46) stimuli. We created relatively low width-to-height ratio (*M* = 2.68, *SD* = 1.74) stimuli (300 × 400 pixels) by cropping 50 pixels from the left and right sides of the illusory face images. This resulted in 50 illusory face images with a relatively high width-to-height ratio and 50 illusory face images with a relatively low width-to-height ratio for the experiment (see Figure 1).

We investigated six dimensions of illusory face perception. To balance the order of questions and mitigate potential order effects, we created six distinct questionnaires, each varying only in the sequence of these questions. All other aspects of the experimental procedure remained consistent across questionnaires. Illusory faces were presented randomly.

At each questionnaire page, participants first viewed an illusory face picture. Below the picture, several questions required them to evaluate the picture using 1–10 Likert scales across the following dimensions: gender: (1 = completely female, 10 = completely male), attractiveness: (1 = not at all attractive, 10 = very attractive), cuteness: (1 = not cute at all, 10 = very cute), trustworthiness: (1 = not at all trustworthy, 10 = very trustworthy), and dominance: (1 = not dominant at all, 10 = very dominant). For emotion evaluation, participants first identified the type of emotion conveyed in the picture by selecting one of the seven options (happy, sad, angry, surprised, scared, disgusted, or neutral) and then rated the intensity of this emotion (1 = a little, 10 = very strong). After completing these questions, participants could proceed to the next page to answer questions about the next illusory face picture.

To ensure participant engagement, each questionnaire included 10 screening questions (e.g., “Please select 1 for this question” or “Please select 10 for this question”). Participants who answered three or more questions incorrectly out of the ten screening questions were considered to have not taken the task seriously, and their data were excluded.

#### 2.1.3. Experimental Design

The experiment employed a within-subjects design. The independent variable was the width-to-height ratio of the illusory face (low, high). The dependent variables were the scores across the following six dimensions: gender, cuteness, trustworthiness, dominance, attractiveness, and emotional perception.

#### 2.1.4. Experimental Procedure

The study was administered via the online platform WJX (www.wjx.cn). To minimize the influence of extraneous variables, participants were instructed to complete the task in a quiet environment in one sitting. All participants completing a specific questionnaire version started at the same time.

Before the formal experiment, participants received instructions to “carefully observe the pictures displayed on each page and score them according to their first impression of gender, attractiveness, cuteness, trustworthiness, dominance (defined as ‘the nature of liking to control other people’s behavior in a powerful way’), and emotion across six dimensions.” Each participant evaluated 100 illusory faces (50 with a high width-to-height ratio, 50 with a low width- to-height ratio), with all dimensions evaluated for each face. The presentation order of the 100 illusory faces was randomized. Participants were informed that they could take breaks during the experiment, not exceeding one minute per break.

#### 2.1.5. Data Analysis

For the mean scores of perceived gender, cuteness, trustworthiness, dominance, and attractiveness (see Table 1), we conducted 2 (participant gender: male, female) × 2 (width- to-height ratio: low, high) multivariate analysis of variance (MANOVA) using SPSS 26 software.

For the mean proportion of emotion type and mean scores of emotion intensity, we conducted 2 (participant gender: male, female) × 2 (width-to-height ratio: high, low) × 7 (emotion type: happy, angry, sadness, surprise, fear, disgust, neutral) ANOVA using SPSS software.

If the sphericity assumption is violated, then the Greenhouse-Geisser correction will be applied. The post hoc tests employ the Bonferroni correction.

### 2.2. Results and Discussion

The 2 (participant gender) × 2 (width-to-height ratio) MANOVA on the perceived gender, cuteness, trustworthiness, dominance, and attractiveness revealed significant univariate effects as follows: In only the attractiveness judgment, there was a significant main effect of facial width-to-height ratio type [*F*(1,52) = 4.881, *p* = 0.032, $\eta_{p}^{2}$ = 0.086] and a significant interaction between it and gender [*F*(1,52) = 4.064, *p* = 0.049, $\eta_{p}^{2}$ = 0.072]. The simple effect test of this interaction showed that high width-to-height ratio faces were rated significantly more attractive than low width-to-height ratio faces only by female participants (*p* = 0.007) but not by male participants (*p* = 0.885). In only the cuteness dimension, there was a main effect of gender [*F*(1,52) = 5.009, *p* = 0.030, $\eta_{p}^{2}$ = 0.088], with females (*M* = 4.727, *SE* = 0.275) rating the faces cuter than males (*M* = 3.903, *SE* = 0.246). All other effects in all other dimensions did not reach significance (*p*s > 0.10).

The 2 (participant gender) × 2 (width-to-height ratio) × 7 (emotion type) ANOVA on the perception of emotion type revealed a significant main effect of emotion type [*F*(3.761,195.577) = 39.084, *p* < 0.001, $\eta_{p}^{2}$ = 0.429]. Participants most frequently chose happiness (*M* = 0.269, *SE* = 0.013), followed by surprise (*M* = 0.192, *SE* = 0.011), neutral (*M* = 0.175, *SE* = 0.014), anger (*M* = 0.120, *SE* = 0.009), sadness (*M* = 0.095, *SE* = 0.008), disgust (*M* = 0.075, *SE* = 0.009), and fear (*M* = 0.073, *SE* = 0.010), while other main effects and interactions were not significant (*p*s > 0.19).

The 2 (participant gender) × 2 (width-to-height ratio) × 7 (emotion type) ANOVA on the emotional intensity ratings revealed a significant main effect of gender [*F*(1,52) = 7.571, *p* = 0.008, $\eta_{p}^{2}$ = 0.132]. Male participants consistently reported stronger emotional intensity (*M* = 6.520, *SE* = 0.257) than female participants (*M* = 5.458, *SE* = 0.288). There was a significant main effect of emotion type [*F*(4.523,226.147) = 3.238, *p* = 0.010, $\eta_{p}^{2}$ = 0.061]. Post hoc comparisons indicated that the intensity of anger (*M* = 6.472, *SE* = 0.290) was significantly greater than that of sadness (*M* = 5.519, *SE* = 0.283) (*p* = 0.001) and neutral (*M* = 5.536, *SE* = 0.229) (*p* = 0.034). No significant intensity differences were found among other emotions (happiness: *M* = 6.171, *SE* = 0.235; surprise: *M* = 6.235, *SE* = 0.222; fear: *M* = 6.106, *SE* = 0.293: disgust: *M* = 5.884, *SE* = 0.306) (*p*s > 0.07). The main effect of width-to-height ratio (*p* = 0.065), the two-way interactions (*p*s > 0.12), and the three-way interaction (*p* = 0.053) did not reach significance.

## 3. Experiment 2: Changing the Shape of the Chin

### 3.1. Methods

#### 3.1.1. Participants

A G*Power 3.1.9.7 (Faul et al., 2009) analysis for a paired *t*-test, with an effect size (*d*) of 0.45, a significance level (α) of 0.05, and a desired statistical power (1 − β) of 0.8, indicated a required sample size of 41 participants. To ensure a balanced experimental design, we initially recruited 42 participants (28 males, 14 females, aged 18–29) through advertisements. All participants voluntarily agreed to participate and received compensation. One participant’s data were excluded due to incorrect responses on three screening questions, resulting in a final sample of 41 participants for data analysis.

#### 3.1.2. Experimental Materials

**Figure 2:**
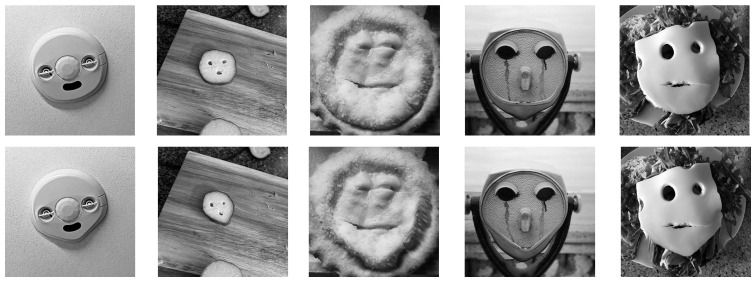
Examples of stimuli in Experiment 2. The images in the top row show illusory faces with a round chin, adapted from Wardle et al. (2022), while the images in the bottom row show illusory faces with a pointed chin, which was created for this study.

A total of 25 illusory face images with round or squared chins from the same source as those in Experiment 1 were selected. All images were manipulated using Photoshop to create the following two sets: 25 illusory faces with round chins and 25 with pointed chins (see Figure 2 for examples). These 50 images served as the formal experimental stimuli. The questionnaire procedure was identical to that used in Experiment 1.

#### 3.1.3. Experimental Design

The experiment employed a within-subject design. The independent variable was the chin shape of the illusory faces (round chin, pointed chin). The dependent variables were the scores across the following six dimensions: gender, cuteness, trustworthiness, dominance, attractiveness, and emotional perception.

#### 3.1.4. Experimental Procedure

The experimental procedure for Experiment 2 was identical to that of Experiment 1, with one exception; in this experiment, participants evaluated 50 illusory faces (25 with round chins and 25 with pointed chins).

#### 3.1.5. Data Analysis

The data analysis is similar to that of Experiment 1, except that the independent variable in this experiment is chin shape (round, pointed).

The participant sample exhibited a notable gender imbalance (male: N = 27; female: N = 14), raising concerns about the validity of the MANOVA analysis, particularly since gender was a factor of interest. Unequal group sizes can compromise the robustness of MANOVA results, especially when key assumptions (e.g., homogeneity of variance–covariance matrices) are not met. To address this, we conducted and reported formal tests of these assumptions.

### 3.2. Results and Discussion

The Box’s M test indicated no significant violation of the homogeneity of variance–covariance matrices assumption [Box’s M = 106.588, *F*(55,2341.597) = 1.289, *p* = 0.078], supporting the appropriateness of the MANOVA procedure.

The 2 (participant gender) × 2 (chin shape) MANOVA on the mean scores (see Table 2) for perceived gender, cuteness, trustworthiness, dominance, and attractiveness revealed no significant main effect of chin shape on dominance judgments [*F*(1,39) = 0.210, *p* = 0.650, $\eta_{p}^{2}$ = 0.005]. However, for gender [*F*(1,39) = 3.788, *p* = 0.059, $\eta_{p}^{2}$ = 0.089], cuteness [*F*(1,39) = 17.836, *p* < 0.001, $\eta_{p}^{2}$ = 0.314], trustworthiness [*F*(1,39) = 5.887, *p* = 0.020, $\eta_{p}^{2}$ = 0.131], and attractiveness [*F*(1,39) = 10.635, *p* = 0.002, $\eta_{p}^{2}$ = 0.214], pointed chin faces were rated as significantly or marginally less favorable than round chin faces. No main effects of participant gender or any interaction effects between participant gender and chin shape were significant across any dimensions (*p*s > 0.42).

The 2 (participant gender) × 2 (chin shape) × 7 (emotion type) ANOVA on the proportion of each emotion type perception showed a significant main effect of emotion type [*F*(3.816,148.840) = 27.467, *p* < 0.001, $\eta_{p}^{2}$ = 0.413]. Participants most frequently selected happiness (*M* = 0.306), followed by surprise (*M* = 0.157, *SE* = 0.013), sadness (*M* = 0.151, *SE* = 0.013), neutral (*M* = 0.141, *SE* = 0.014), anger (*M* = 0.107, *SE* = 0.010), fear (*M* = 0.081, *SE* = 0.017), and disgust (*M* = 0.057, *SE* = 0.011). This distribution of perceived emotion type differed from that found in Experiment 1, likely due to variations in the specific illusory face stimuli used between the two experiments. No other main effects or interactions were significant (*p*s > 0.30).

The 2 (participant gender) × 2 (chin shape) × 7 (emotion type) ANOVA on the emotional intensity ratings for the chosen emotions revealed a significant main effect of emotion type [*F*(3.801,148.248) = 8.370, *p* < 0.001, $\eta_{p}^{2}$ = 0.177]. The intensity ratings were strongest for happiness (*M* = 6.464, *SE* = 0.311), anger (*M* = 6.299, *SE* = 0.374), sadness (*M* = 6.276, *SE* = 0.355), and surprise (*M* = 6.141, *SE* = 0.267), followed by disgust (*M* = 4.804, *SE* = 0.488), neutral (*M* = 4.532, *SE* = 0.373), and fear (*M* = 4.465, *SE* = 0.409). Other main effects and two-way interactions were not significant (*p*s > 0.46).

## 4. Experiment 3: Changing the Distance Between Eyes and Mouth

### 4.1. Methods

#### 4.1.1. Subjects

A G*Power 3.1.9.7 (Faul et al., 2009) analysis for a repeated measure ANOVA, with an effect size (*d*) of 0.45, a significance level (α) of 0.05, and a desired statistical power (1 − β) of 0.8, indicated a required sample size of 30 participants. This calculation assumed three groups, 15 measurements per group, and a correlation between repeated measurements of 0.5. To ensure a balanced experimental design, we recruited a total of 60 participants (23 males, 37 females, aged 19–24) via advertisements. All participants voluntarily agreed to participate and received compensation.

#### 4.1.2. Experimental Materials

**Figure 3:**
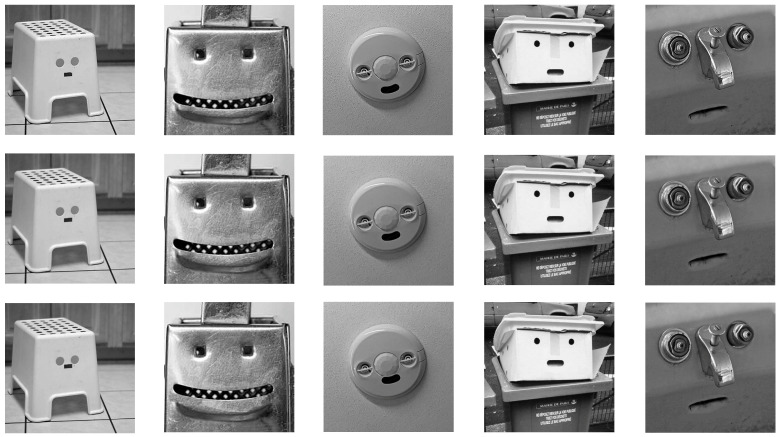
Examples of stimuli demonstrating varying eye–mouth distances in Experiment 3. The images in the top row show illusory faces with a large eye–mouth distance, created for this study; the images in the middle row depict illusory faces with an original eye–mouth distance, adapted from Wardle et al. (2022); and the images in the bottom row illustrate illusory faces with a small eye–mouth distance, also created for this study.

The 15 illusory face images used in this experiment were sourced from the same collection as those in Experiment 1. We manipulated these images using Photoshop to create variations in eye–mouth distance. The mouths of the original illusory faces were moved up by 10 pixels to create images with a small eye–mouth distance, and they were moved down by 10 pixels to create images with a large eye–mouth distance. The unmodified original images served as the normal eye–mouth distance condition. This resulted in three sets of 15 illusory faces each (large, original, and small eye–mouth distances), totaling 45 experimental stimuli (see Figure 3 for examples). The questionnaire procedure was identical to that used in Experiment 1.

#### 4.1.3. Experimental Design

The experiment used a within-subject design. The independent variable was the eye–mouth distance of the illusory face (large, original, small). The dependent variables were the scores across the following six dimensions: gender, cuteness, trustworthiness, dominance, attractiveness, and emotional perception.

#### 4.1.4. Experimental Procedure

The experimental procedure for Experiment 3 was identical to that of Experiment 1, with one exception, which was the stimuli. In this experiment, participants evaluated 45 illusory faces, consisting of 15 with large eye–mouth distances, 15 with small eye–mouth distances, and 15 original illusory faces.

#### 4.1.5. Data Analysis

The data analysis is similar to that of Experiment 1 except that the independent variable in this experiment is eye–mouth distance (large, original, small).

### 4.2. Results and Discussion

The 2 (participant gender) × 3 (eye–mouth distance) MANOVA on the mean scores (see Table 3) for perceived gender, cuteness, trustworthiness, dominance, and attractiveness revealed no significant main effects or interaction effects across any of the dimensions (*p*s > 0.18).

The 2 (participant gender) × 3 (eye–mouth distance) × 7 (emotion type) ANOVA on the proportion of each participant’s chosen emotion type showed a significant main effect of emotion type [*F*(3.717,215.564) = 22.821, *p* < 0.001, $\eta_{p}^{2}$ = 0.282]. Participants most frequently selected neutral (*M* = 0.252, *SE* = 0.022), followed by surprise (*M* = 0.211, *SE* = 0.015), sadness (*M* = 0.164, *SE* = 0.015), happiness (*M* = 0.150, *SE* = 0.014), fear (*M* = 0.093, *SE* = 0.011), anger (*M* = 0.069, *SE* = 0.009), and disgust (*M* = 0.061, *SE* = 0.008). Other main effects and interactions were not significant (*p*s > 0.28). This distribution of perceived emotion type differed from that found in Experiments 1 and 2, likely due to variations in the specific illusory face stimuli used between these experiments.

The 2 (participant gender) × 3 (eye–mouth distance) × 7 (emotion type) ANOVA on the emotional intensity ratings revealed a significant main effect of gender, with male participants (*M* = 5.247, *SE* = 0.245) consistently reporting stronger emotional intensity than female participants (*M* = 4.163, *SE* = 0.193). A significant main effect of emotion type was also found [*F*(4.553,264.077) = 7.971, *p* < 0.001, $\eta_{p}^{2}$ = 0.121]. The intensity ratings were strongest for surprise (*M* = 5.948, *SE* = 0.207), sadness (*M* = 5.352, *SE* = 0.289), and neutral (*M* = 5.124, *SE* = 0.257) emotions, followed by happiness (*M* = 4.781, *SE* = 0.336), fear (*M* = 4.369, *SE* = 0.350), anger (*M* = 3.848, *SE* = 0.383), and then disgust (*M* = 3.512, *SE* = 0.388). Other main effects or interactions were not significant (*p*s > 0.16).

## 5. General Discussion

This study takes an initial step in investigating how manipulating the width-to-height ratio, chin shape, and eye–mouth distance of illusory faces influences their perceived gender, cuteness, trustworthiness, dominance, attractiveness, and emotion. Our findings reveal some similarities and differences in the influence of facial features on illusory faces compared to real faces, with notable gender differences in some perceptions.

### 5.1. Width-to-Height Ratios (Experiment 1)

Experiment 1 demonstrated that high width-to-height ratios enhanced attractiveness, an effect observed exclusively among female participants. This suggests a potential gender-specific processing route for attractiveness in illusory faces. One plausible interpretation is that the illusory faces were generally perceived as male in the present study (mean gender judgment > 5.5) and in Wardle et al. (2022), potentially explaining why female participants rated these faces as more attractive. This is because female participants tend to prefer masculine men (Docherty et al., 2020).

Our results regarding width-to-height ratio’s impact on dominance, gender perception, and trustworthiness differ from findings in the real face literature. For instance, Carré et al. (2009) found that higher width-to-height ratios in real faces correlated with increased dominance, and Stirrat and Perrett (2010) reported that higher width-to-height ratios in male faces were associated with lower trustworthiness. This discrepancy might stem from the unique characteristics of illusory faces. The average width-to-height ratio of illusory faces (3.71) used in Wardle et al. (2022) is substantially higher than that of real human faces (1.3–2.4, Durkee & Ayers, 2021). Even our “low” width-to-height ratio (*M* = 2.68, *SD* = 1.74) stimuli remained relatively high, potentially leading participants to consistently perceive them as male (mean gender judgment > 5.5). This inherent “maleness” of illusory faces, regardless of width-to-height ratio manipulation, could have muted the impact of width-to-height ratio on perceived gender and dominance in ways observed with real faces.

### 5.2. Chin Shape (Experiment 2)

Experiment 2 highlighted the significant role of chin shape in the social evaluation of illusory faces. Specifically, round chins, compared to pointed chins, were perceived as showing more maleness and being cuter, more trustworthy, and more attractive. This effect did not extend to dominance judgments.

These findings strongly align with the existing literature on real faces. The perception of round chins as more masculine is consistent with anthropological observations that men generally have wider chins, while women tend to have more pointed chins (Bass & Riggio, 2006; Byers, 2016). This also resonates with Wardle et al.’s (2022) finding that illusory faces are frequently perceived as male; our results suggest that a contributing factor to this phenomenon is likely because the objects forming these illusory faces tend to have square or round chins rather than pointed ones. Similarly, the enhanced cuteness of round chins is consistent with studies on “baby schema” (Almanza-Sepúlveda et al., 2018; Glocker et al., 2009), where rounder facial features are associated with higher cuteness. These consistencies suggest that the influence of chin shape on social perceptions extends effectively from real faces to illusory faces.

### 5.3. Eye–Mouth Distance (Experiment 3)

In contrast to width-to-height ratio and chin shape, Experiment 3 found that eye–mouth distance did not significantly affect any of the perceived characteristics of the illusory faces (gender, cuteness, trustworthiness, dominance, attractiveness, or emotion type). This result runs counter to the literature on real faces, which suggests that a smaller eye–mouth distance can enhance cuteness and attractiveness (Glocker et al., 2009) and improve credibility (Hu, 2024). The lack of broad effects for eye–mouth distance in illusory faces might be due to several factors, as discussed in the limitations.

### 5.4. Perceived Emotion Type and Intensity

Across three experiments, we found no significant evidence that manipulating the width-to-height ratio, chin shape, or eye–mouth distance of illusory faces influenced participants’ perceptions of emotion type or intensity. However, consistent gender differences emerged in Experiments 1 and 3; male participants reported significantly stronger overall emotional intensity than female participants. This finding appears to conflict with Proverbio and Galli (2016), who demonstrated that women’s brains are more prone to anthropomorphizing objects, with face–object perception in women activating areas related to emotional and social information processing. In contrast, men showed greater activation in areas linked to shape processing. These discrepancies highlight the need for further research into gender differences in the emotional perception of illusory faces.

### 5.5. Limitations and Future Directions

Our study has several limitations that warrant consideration for future research. A significant concern raised by participants was the perceived lack of sensitivity in the rating task to the subtle differences between manipulated illusory face images. Participants reported an impression of “repeated pictures,” potentially leading them to recall prior ratings rather than evaluating each manipulated image freshly. This issue was particularly prominent in Experiments 1 and 3, which involved subtle manipulations. This could have diminished the power to detect significant differences. Future studies should consider using forced-choice tasks, which may be less susceptible to such recall biases, and should conduct pre-experimental verification of stimulus distinctiveness across manipulated dimensions.

Additionally, while our experimental materials originated from established studies, the final selection of specific images was subjective, and the limited number of stimuli per manipulation condition may restrict the generalizability of our findings. Future research should employ a larger and more diverse set of stimuli to enhance the robustness and broader applicability of the results. Ideally, all manipulated pictures should undergo pre-experimental testing to ensure the intended perceptual effects before the main experiment.

It should be noted that, since the stimuli are all natural pictures, the illusory faces may be oriented at different angles. As a result, the width-to-height ratio that we measured may not be absolutely accurate. However, we do not believe that the angle of rotation of the stimuli will affect the results. This is because the manipulation of independent variables, such as the width-to-height ratio, occurs within the images themselves. For example, both high and low width-to-height ratio images have the same angle of rotation.

## 6. Conclusions and Implications

In summary, this study provides valuable insights into the social perception of illusory faces, demonstrating that their attractiveness is influenced by width-to-height ratio (especially for females) and that chin shape significantly impacts perceptions of masculinity, cuteness, trustworthiness, and attractiveness. Our findings extend the applicability of social evaluation principles from real faces to illusory faces, particularly concerning chin shape.

These research results have significant implications for product design and marketing. Designers can leverage these findings by creating illusory face elements in products, packaging, or advertisements that feature higher width-to-height ratios and round chins to enhance attractiveness and stimulate consumer interest. This could optimize user experience, making products appear more friendly and appealing. Furthermore, in the realm of virtual humanoids, such as companion robots, designing trustworthy and attractive virtual faces based on these principles could foster stronger human–machine partnerships.

Finally, this study offers a new perspective for future research by highlighting gender differences in the social evaluation of illusory faces. Future investigations into misperceived faces should explicitly test for gender-specific effects, recognizing that significant findings may be contingent on participant gender.

## Figures and Tables

**Table 1:** Means and standard deviations of perceived gender, cuteness, trustworthiness, dominance, and attractiveness of illusory faces, categorized by participant gender and facial width-to-height ratio (WHR).

Dimension	Male Participants (*N* = 30)		Female Participants (*N* = 24)	
	High WHR	Low WHR	High WHR	Low WHR
Gender	5.86 (0.72)	5.90 (0.72)	5.89 (1.12)	5.91 (1.15)
Cuteness	3.94 (1.44)	3.87 (1.32)	4.73 (1.30)	4.72 (1.33)
Trustworthiness	5.41 (1.14)	5.47 (1.17)	5.01 (1.21)	4.99 (1.19)
Dominance	5.50 (1.63)	5.53 (1.63)	4.77 (1.51)	4.83 (1.56)
Attractiveness	4.75 (1.56)	4.75 (1.56)	4.90 (1.37)	4.71 (1.50)

**Table 2:** Means and standard deviations of perceived gender, cuteness, trustworthiness, dominance, and attractiveness of illusory faces, categorized by participant gender and chin shape.

Dimension	Male Participants (*N* = 27)		Female Participants (*N* = 14)	
	Pointed Chin	Round Chin	Pointed Chin	Round Chin
Gender	5.62 (1.04)	5.76 (0.93)	5.61 (0.46)	5.72 (0.48)
Cuteness	4.88 (1.37)	5.13 (1.26)	4.99 (1.57)	5.26 (1.57)
Trustworthiness	5.40 (1.34)	5.54 (1.21)	5.13 (1.54)	5.28 (1.54)
Dominance	5.50 (1.59)	5.55 (1.44)	5.12 (1.53)	5.13 (1.57)
Attractiveness	5.33 (1.59)	5.57 (1.48)	5.09 (1.48)	5.26 (1.49)

**Table 3:** Means and standard deviations of perceived gender, cuteness, trustworthiness, dominance, and attractiveness of illusory faces, categorized by participant gender and eye–mouth distance.

Dimension	Male Participants (*N* = 23)			Female Participants (*N* = 37)		
	Large	Original	Small	Large	Original	Small
Gender	5.57 (0.89)	5.57 (0.87)	5.56 (0.62)	5.86 (0.88)	5.87 (0.92)	5.84 (1.00)
Cuteness	4.52 (1.27)	4.64 (1.39)	4.59 (1.28)	4.53 (1.35)	4.44 (1.27)	4.39 (1.25)
Trustworthiness	4.58 (1.54)	4.74 (1.56)	4.63 (1.50)	4.65 (1.47)	4.68 (1.41)	4.60 (1.38)
Dominance	4.59 (1.93)	4.56 (2.13)	4.52 (2.00)	4.39 (1.52)	4.37 (1.54)	4.37 (1.52)
Attractiveness	4.63 (1.51)	4.64 (1.43)	4.63 (1.47)	4.43 (1.25)	4.49 (1.27)	4.42 (1.17)

## Data Availability

The data presented in the study are openly available in OSF at https://osf.io/2z6ws/ (accessed on 10 July 2025).
